# Myeloid/lymphoid neoplasm with eosinophilia and BCR/FGFR1 rearrangement with transformation to cortical T-lymphoblastic lymphoma and erythroid precursors: a case report

**DOI:** 10.1186/s13256-022-03722-y

**Published:** 2023-01-26

**Authors:** Alejandro Pineda Isaza, Santiago Castaño Quintero, Lisceth Paola Quintero González, Fabián Emiliano Ahumada Córdoba, Andrés Felipe Arbeláez Olivar, Juan Carlos Bravo Ocaña

**Affiliations:** 1 Health Sciences Department, Universidad Icesi, Cali, Colombia; 2 Haemato-Oncology Service, Internal Medicine Department, Fundación Valle del Lili, Cali, Colombia; 3 Pathology Service, Pathology and Laboratory Department, Fundación Valle del Lili, Cali, Colombia

**Keywords:** Myeloid/lymphoid neoplasms, Precursor T-cell lymphoblastic leukemia–lymphoma, Eosinophilia, FGFR1 rearrangement

## Abstract

**Background:**

Myeloproliferative neoplasms are a group of diseases with diverse biological and clinical characteristics. As a provisional separate entity, myeloid/lymphoid neoplasms with eosinophilia and genetic rearrangement have been described, which may present an initial clinical behavior of myeloproliferation and be characterized by varied genetic rearrangements. One of these entities is associated with FGFR1 rearrangements, characterized by its low prevalence and few treatment options.

**Case presentation:**

We present the case of a 53-year-old Mestizo male patient of Hispanic origin who initially presented weight loss and fatigue, with a complete blood count showing leukocytosis and eosinophilia, with an initial diagnosis of nonspecific myeloproliferative disorder. In a next-generation sequencing study, BCR::FGFR1 rearrangement was documented, a diagnosis of myeloid/lymphoid neoplasia with eosinophilia and BCR::FGFR1 rearrangement was made, and hydroxyurea therapy was initiated. Subsequently, transformation to cortical T-lymphoblastic leukemia/lymphoma and erythroid precursors was documented, requiring management with chemotherapy.

**Conclusions:**

Myeloid/lymphoid neoplasms with eosinophilia and genetic rearrangements constitute a group of deeply heterogeneous diseases with variable clinical and diagnostic characteristics and whose treatment is not clearly defined.

## Introduction

Myeloproliferative neoplasms (MPN) are clonal hematopoietic disorders characterized by proliferation of one or more cellular lineages: myeloid, erythroid, granulocytic, or megakaryocytic [1]. These disorders phenotypically mimic each other, as with myeloid neoplasms and even benign hematopoietic disorders. Each myeloproliferative neoplasm can evolve to another one, which confuses the diagnosis, risk assessment, and therapeutic options [2]. Since William Dameshek first described five subgroups of myeloproliferative diseases, until the World Health Organization (WHO) established the current classification in 2016, multiples changes have been made given further knowledge about molecular pathways in disease, including new entities with different clinical and genetic characteristics [3]. We present the case of a male adult patient diagnosed with myeloid/lymphoid neoplasm with eosinophilia and BCR::FGFR1 rearrangement with transformation to cortical T-lymphoblastic lymphoma and erythroid precursors.

## Case presentation

We report the case of a male 53-year-old Mestizo patient, without medical record. He initially presented to the emergency department in March 2019 because of 12 kg weight loss for 6 months, fatigue, and one syncopal event. He arrived to the emergency room, where tests were carried out. His blood cell count revealed leukocytes 40 × 10^9^/L, neutrophils 24 × 10^9^/L, lymphocytes 12 × 10^9^/L, eosinophils 3.5 × 10^9^/L, hemoglobin 12.6 g/dL, and platelets 120 × 10^9^/L, without other alterations. A bone marrow biopsy plus aspiration was carried out to assess myeloid proliferation, which revealed no myeloid or lymphoid blasts. The morphological and immunohistochemical characteristics revealed in this test suggest a chronic myeloproliferative neoplasm with an unspecific histological pattern, as well as a mild increase of reticular network.

Other studies were performed between April and August 2019, including: karyotype 46XY (normal), JAK2 V617F, unmutated CALR exon 9, and MPL exon 10. Assessed in hematology consult in December 2019, new blood cell count showed leukocytes 14.8 × 10^9^/L, neutrophils 6.5 × 10^9^/L, monocytes 1.65 × 10^9^/L, eosinophils 1.31 × 10^9^/L, hemoglobin 19.3 g/dL, hematocrit 60%, and platelets 41 × 10^9^/L. A next-generation sequencing test was requested to identify other mutations. In February 2020, a fusion between BCR::FGFR1 was described, which corresponds to BCR::FGFR1 *t*(8;22) (p11;q11) (chr8:38418680::chr22:23239452). This result is concordant with myeloid/lymphoid neoplasm with eosinophilia and BCR::FGFR1 rearrangement; therefore, cytoreductive treatment with hydroxyurea and phlebotomies were started according to cytopenia and/or cytosis. In September 2021, he had anemia with hemoglobin of 5.4 g/dL. Again, bone marrow study revealed no blasts and showed hypercellularity with myeloid predominance and reticulin fibrosis grade 2. The patient also had splenomegaly, 20 cm spleen without other echogenicity impairments, and splenic hilum adenomegalies. Hydroxyurea was suspended, and medical surveillance was continued.

Owing to refractory cytopenia and the presence of perisplenic lymphadenopathy, splenectomy and surgical resections of adenomegalies were performed on 9 March 2022; subsequently, the cytopenia improved.

**Fig. 1:**
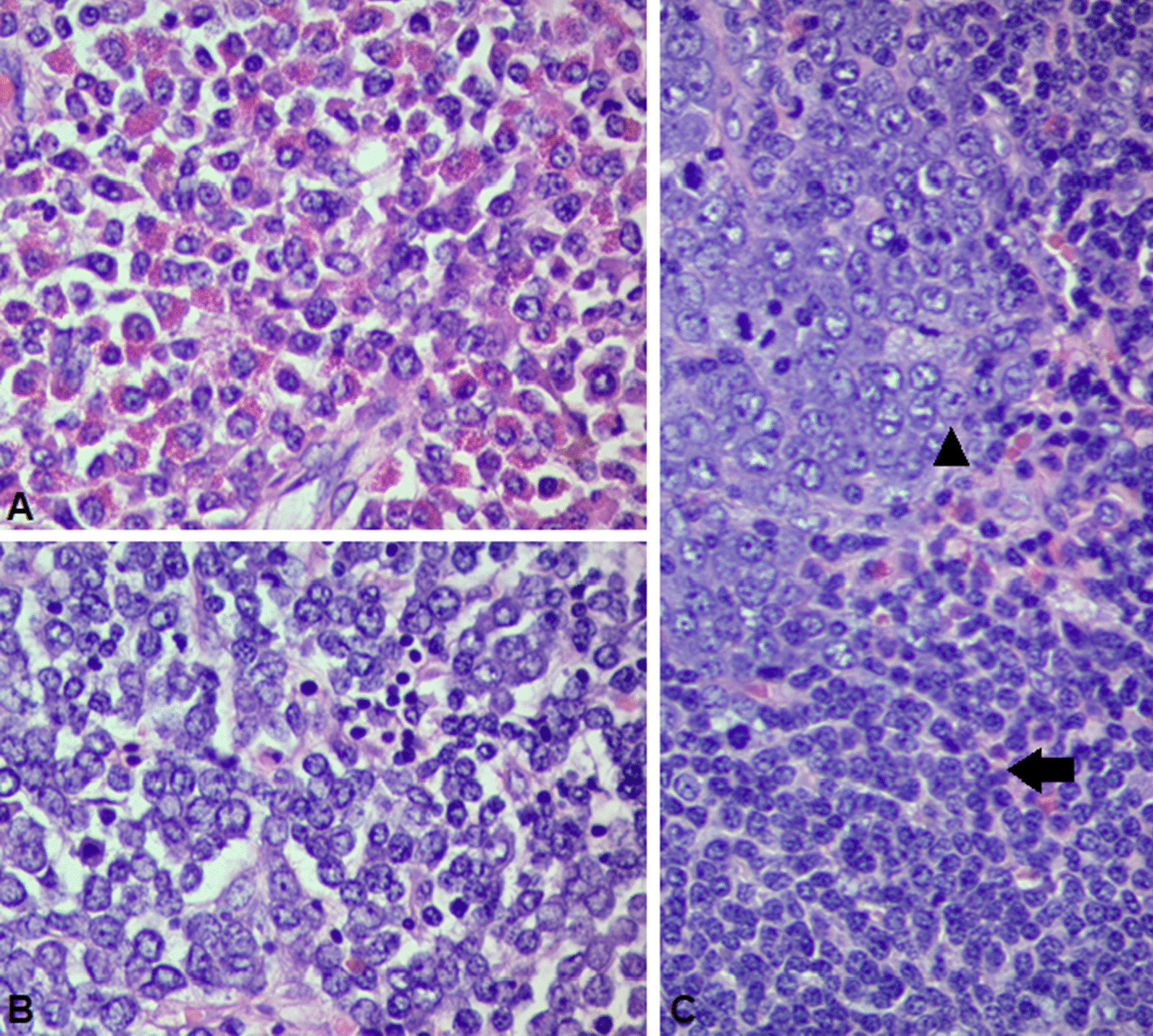
Splenic tissue samples demonstrating cellular proliferation with a histological pattern that has a distribution of sheets of mature eosinophils with its precursors and are accompanied with other big cells distributed in nests, with immature chromatin nuclei, nucleoles, and scarce cytoplasm (**A** and **B**). Lymphatic node samples evidencing infiltration by immature cells described previously at the spleen, which comprise immature chromatin nuclei with abundant eosinophils and mastocytes (arrowhead). Furthermore, different lymph node zones are infiltrated by lymphoblastic cells with diffuse growth pattern (arrow) (**C**)

**Fig. 2:**
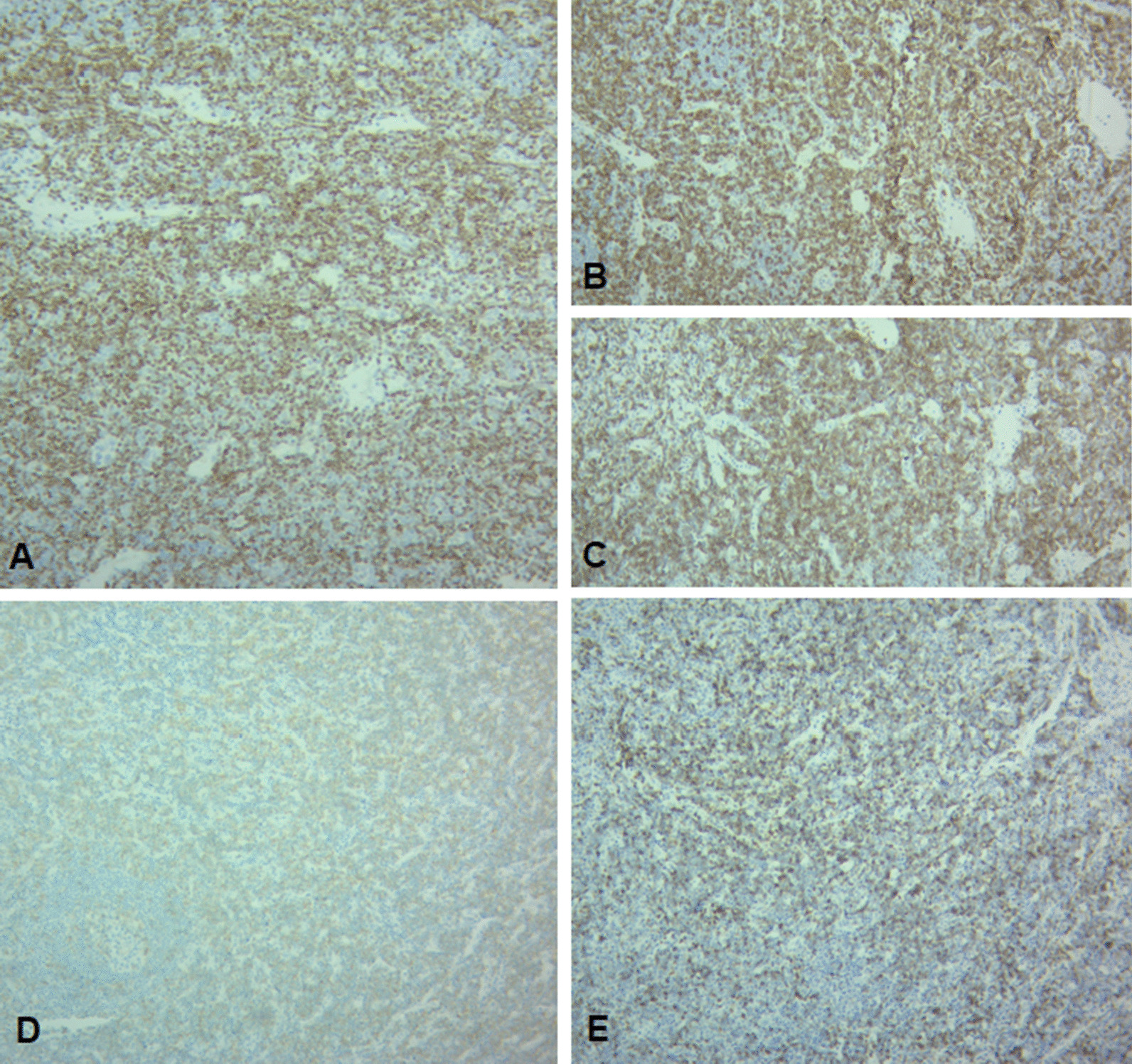
Population infiltrating the node corresponds to cortical T lymphoblasts, which are positive for TdT (**A**), CD1a (**B**), CD3 (**C**), CD4, CD8, CD99, CD5, CD7, and BCL2. Neoplastic population of positive erythroid precursors is found positive for E-cadherin (**D**), glycophorin (**E**), and heterogeneous weak expression of CD117. The cellular proliferation index calculated with ki67 is 95%

The biopsy documented infiltration by cortical T-cell lymphoblastic lymphoma associated with erythroid precursors and eosinophilia (Figs. 1 and 2).

Positron emission tomography–computed tomography (PET–CT) with 18-FDG (18F-fluorodeoxyglucose) was performed to stage the disease, revealing a bone marrow with heterogeneous metabolic hyperactivity. This finding suggests neoplastic infiltration and lytic and blastic lesions located in most of bone structures of axial and proximal appendicular skeleton. PETHEMA LAL-AR 2011 protocol was started, without any adverse events, with subsequent complete metabolic response according to new PET–CT, complete hematological response, and negative measurable minimal disease. Human leucocyte antigen (HLA) studies were requested to consolidate with allogeneic hematopoietic stem cell transplantation. The patient is currently receiving a consolidation scheme of the chemotherapy protocol.

## Discussion

Myeloid/lymphoid neoplasms with eosinophilia and genetic rearrangements constitute a group of diseases, initially described by McDonald *et al*. in 1995, when they identified a patient with MPN, eosinophilia in bone marrow, and T-lymphoblastic lymphoma with documented balanced translocation 8p11 [4].

Subsequently, other genetic rearrangements were described with genes including *PDGFRA*, *PFDGFRB*, *FGFR1*, and *PCM1-JAK2*. The myeloid/lymphoid neoplasms with eosinophilia and FGFR1 rearrangements are a provisional entity according to 2016 WHO classification, defined as a heterogeneous neoplasm, consequence of multiple translocations with a breaking point 8p11. Depending on the associated chromosome at the translocation, a variety of fusion genes may be shaped, thus incorporating part of FGFR1. All these fusion genes code for an aberrant tyrosine kinase [5]. The most common clinical manifestations are fatigue, pruritus, hepatosplenomegaly, and impairments associated with eosinophilia, including pulmonary fibrosis, restrictive myocardiopathy, eosinophilic gastritis, and/or dermatitis. In this case, we documented severe eosinophilia, without impaired organ or splenomegaly. Elevated tryptase levels in serum and elevated vitamin B12 with granulocytic hyperplasia have been reported frequently [6]. The cases may present as MPN or transforming acute myeloid leukemia, T- or B-lymphoblastic leukemia/lymphoma, or mixed-phenotype acute leukemia [5]. Using murine models, some authors developed a bone marrow transduction and transplantation approach to develop a model for FGFR1OP2-FGFR1 disease, documenting the appearance of CD4^+^ T-cell lymphoblastic lymphoma and acute myeloid leukemia [7]. A systematic review and meta-analysis of the literature included 20 cases of patients with myeloproliferative neoplasms with transformation to the leukemic phase who were carriers of the BCR::FGFR1 fusion *t*(8;22) (p11;q11). Sixty percent had B acute lymphoblastic leukemia, 26.7% leukemia of undetermined lineage, 6.7% acute lymphoblastic leukemia of B/T phenotype, and 6.7% acute leukemia of mixed myeloid/T phenotype [8]. FGFR1 fusions could lead to downstream signaling through the PI3K/AKT and RAS/MAPK pathways, which can lead to growth and survival of neoplastic cells [9].

The diagnostic criteria are determined by documentation of a MNP or myelodysplastic/myeloproliferative neoplasm with prominent eosinophilia and in some cases neutrophilia or monocytosis, or acute myeloid leukemia, or mixed-phenotype T- or B-lymphoblastic leukemia/lymphoma, and the presence of *t*(8;13) (p11;q12), or a variant translocation leading to FGFR1 rearrangements, evidenced in myeloid cells, lymphoblasts or both [10]. In this case, the gene fusion BCR::FGFR1 corresponding to BCR::FGFR1 *t*(8;22) (p11;q11), was documented through next-generation sequencing.

Different therapeutic approaches have been used, documented in case series and retrospective studies, based on high-intensity chemotherapy such as hyper-CVAD for B or T lymphomas/leukemias and cytarabine or hydroxyurea for acute myeloid leukemias; the results are heterogeneous, most of them with global survival of less than 2 years [4].

In this case, a chemotherapy protocol based on vincristine, l-asparaginase, daunorubicin, and dexamethasone was started. The treatment with tyrosine-kinase inhibitor pemigatinib, which inhibits FGFR1, FGFR2, and FGFR3, has been assessed in a phase II trial, verifying an objective global response of 85% [10]. Nowadays, the single curative therapy is the consolidation with allogeneic hematopoietic stem cell transplantation. In a case series where 22 patients diagnosed with myeloid/lymphoid neoplasm associated with eosinophilia and FGFR1 rearrangements were treated with allogeneic hematopoietic stem cell transplantation, 7 of them had BCR::FGFR1 rearrangement. After a 4.1-year median follow-up, 32% had died. The survival median had not been reached, and estimated survival rate at 1, 3, and 5 years was 77%, 74%, and 74%, respectively.

All the patients who were transplanted during the untransformed myeloproliferative phase were alive at the last control. The death causes were relapse/progression (18%), graft versus host disease (9%), and multiorgan failure/toxicity (4.5%). [11]

## Conclusions

Myeloid/lymphoid neoplasms with eosinophilia and genetic rearrangements constitute a group of deeply heterogeneous diseases, with variable clinical and diagnostic characteristics, and whose treatment is not clearly defined. The subgroup of entities, composed by FGFR1 rearrangements, has a characteristic poor short-term prognosis and a high probability to transform to acute leukemias. The treatment with tyrosine-kinase inhibitors has documented an adequate objective global response, but further studies are required. Until now, the treatment is based on the disease’s behavior at the time of transformation, using combined chemotherapy regimens depending on the compromised cellular lineage. The evidence is predominantly based on case reports documenting effectivity and safety in different clinical scenarios. Higher-quality studies are required to lead the approach of these patients.

## Data Availability

Not applicable.
